# Infecção pelo SARS-Cov-2 e Tromboembolismo Pulmonar – Comportamento Pró - Trombótico da COVID-19

**DOI:** 10.36660/abc.20200427

**Published:** 2020-07-28

**Authors:** Hellen Dutra Passos, Mariana Carvalho Alves, Leonardo Baumworcel, João Paulo Cerqueira Vieira, Juliane Dantas Seabra Garcez, Antônio Carlos Sobral Sousa

**Affiliations:** 1 Clínica e Hospital São Lucas/Rede D’Or AracajuSE Brasil Clínica e Hospital São Lucas/Rede D’Or, São Luiz, Aracaju, SE - Brasil; 2 Divisão de Cardiologia Hospital Universitário Universidade Federal de Sergipe São CristovãoSergipe Brasil Divisão de Cardiologia do Hospital Universitário da Universidade Federal de Sergipe,São Cristovão, Sergipe, Brasil; 3 Departamento de Medicina Universidade Federal de Sergipe São CristovãoSE Brasil Departamento de Medicina da Universidade Federal de Sergipe, São Cristovão, SE - Brasil; 4 Programa de Pós-Graduação em Ciências da Saúde Universidade Federal de Sergipe São CristovãoSergipe Brasil Programa de Pós-Graduação em Ciências da Saúde da Universidade Federal de Sergipe, São Cristovão, Sergipe, Brasil

**Keywords:** Coronavirus, COVID-19, Embolia Pulmonar, Síndrome Respiratória Aguda Grave, Anticoagulantes, Diagnóstico por Imagem

## Introdução

A COVID-19, causada pelo novo coronavírus, o qual foi batizado pela OMS de “SARS-CoV-2” (sigla em inglês para *Severe Acute Respiratory Syndrome CoronaVirus* 2) espalhou-se pelo planeta com uma velocidade surpreendente.^1^ No Brasil, o seu risco de alastramento (R_0_) tem sido de aproximadamente 3.0, o que explica a sua rápida disseminação por todos os Estados da União.^2^

Portadores de doenças cardiovasculares, hipertensão arterial sistêmica, diabetes mellitus, doença pulmonar obstrutiva crônica e os imunodeprimidos têm maior probabilidade de desfechos adversos.^3^

Uma incidência relativamente alta de doença trombótica e tromboembólica tem sido observada em portadores de COVID-19, provavelmente decorrente de efeitos diretos do SARS-CoV-2 ou por mecanismos indiretos da própria infecção. Ainda, interações medicamentosas entre terapias contra COVID-19 e agentes antiplaquetários ou anticoagulantes, e a interrupção inadvertida de drogas anticoagulantes podem, também, contribuir para o estado pró-trombótico encontrado nesta doença.^4^

## Objetivos

Descrição de caso de paciente diagnosticado com infecção pelo SARS-CoV-2 evoluindo com tromboembolismo pulmonar sem evidência de trombose periférica.

## Métodos

As informações contidas neste relato clínico foram obtidas mediante revisão de prontuário eletrônico, registro de exames complementares e revisão de literatura.

## Relato de Caso

**Figura 1:**
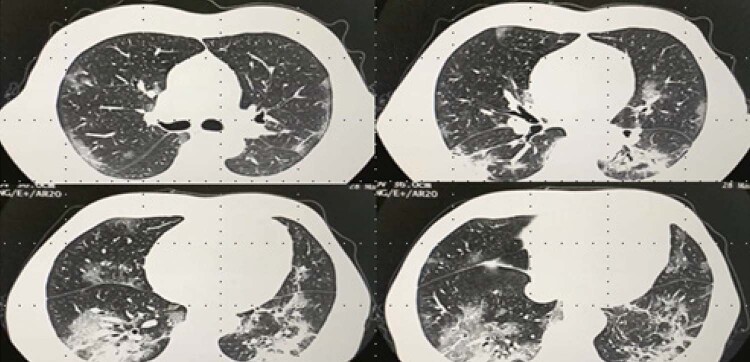
– *Tomografia de tórax sem contraste da admissão hospitalar: áreas com atenuação em vidro fosco, predominando em periferia, sendo mais evidente em segmentos inferiores, acometendo menos que 50% do parênquima pulmonar.*

Paciente I.M.S., do sexo masculino, de 66 anos, procurou o setor de urgência de um hospital geral em Aracaju – SE, Brasil, em 28/03/2020, com quadro de congestão nasal, tosse seca, astenia, náusea e febre (chegando a 40º C), iniciados há oito dias, que havia se intensificado nas últimas 48 horas. Referiu ter realizado implantes dentários na cidade do Rio de Janeiro no início de março e retornado a Aracaju dia 18/03/2020. Nega comorbidades significativas, alergias ou uso regular de medicamentos. Cirurgia prévia de osteossíntese de úmero esquerdo há 16 anos, ex-tabagista (cessou há > 10 anos), é praticante regular, de atividade física. Ao exame físico, chamava a atenção apenas a presença de roncos difusos e sibilos inspiratórios na ausculta pulmonar. Encontrava-se eupneico, acianótico e estável do ponto de vista hemodinâmico. Foi, então, aventada a hipótese de COVID-19 e solicitados: 1) exames laboratoriais de rotina, evidenciando-se apenas proteína C reativa discretamente elevada, com demais parâmetros dentro da normalidade (incluindo marcadores de lesão miocárdica); 2) RT-PCR por swab orofaríngeo; e 3) realizada tomografia do tórax ( Figura 1 ) que evidenciou áreas com atenuação em vidro fosco, predominando em periferia e sendo mais evidente em segmentos inferiores, acometendo menos que 50% do parênquima pulmonar. O eletrocardiograma (ECG) foi considerado dentro dos parâmetros de normalidade, com ritmo sinusal e frequência cardíaca de 65bpm.

Por se tratar de idoso com acometimento pulmonar, o paciente foi internado em isolamento, na Unidade de Terapia Intensiva, com o diagnóstico de pneumonia viral, provavelmente por SARS-CoV-2.

Foi iniciada terapia com Oseltamivir 150mg/dia, Azitromicina 500mg/dia e Ceftriaxona 2g/dia e associado profilaxia para trombose venosa com enoxaparina 40mg/dia. Após 24h de internação, o paciente evoluiu com piora progressiva do padrão respiratório, culminando em insuficiência respiratória aguda, sendo necessária a intubação orotraqueal no segundo dia de hospitalização. Apresentou, também, quadro clínico de choque, recorrendo ao uso de droga vasoativa.

Diante da piora hemodinâmica e com o resultado da RT-PCR negativo para SARS-CoV-2, foi solicitado um ecocardiograma transesofágico (ETE) para afastar endocardite infecciosa. O ETE, realizado dia 02/04/2020, revelou: a) aumento do ventrículo esquerdo, o qual apresentava hipocinesia difusa predominantemente da parede ântero-apical e septo apical, disfunção diastólica grau 1 e disfunção sistólica global de grau moderada, com fração de ejeção de 41%; b) aumento de câmaras direitas, com hipocinesia difusa do ventrículo direito, e disfunção sistólica global de grau moderado pela análise subjetiva; c) hipertensão pulmonar de grau leve a moderado (pressão sistólica em artéria pulmonar, estimada em 48 mmHg); d) alterações degenerativas das valvas aórtica e mitral; e) ausência de vegetações e/ou trombos. Foi então levantada a hipótese de miopericardite, todavia, o ECG e os marcadores de lesão miocárdica eram normais. A ressonância magnética do coração, de grande valia nestas circunstâncias, foi postergada em decorrência do quadro infeccioso vigente.

Posteriormente, constatou-se elevação exponencial do dímero-D plasmático e da proteína C reativa, enquanto os níveis de troponinas e do NT-pró-BNP permaneciam dentro das faixas normais, conforme pode ser apreciado na Tabela 1 . Vale ressaltar que o resultado negativo do RT-PCR pode ser explicado, em parte, porque a amostra para o teste foi colhida no nono dia do início dos sintomas, quando, sabidamente, a eliminação viral está em queda. Tendo em vista, forte suspeita de COVID-19, foi mantido o esquema de antibiótico e solicitado teste sorológico, cujo resultado foi positivo para a doença (IgG - / IgM + SARS-Cov-2).

**Tabela 1:** – Evolução dos marcadores durante o período de internação

Tempo de Internação	D-dímero (ug)	Troponina (ug/ml)	NT - ProBNP (ug/ml)
1º dia	288	<0,012	-
2º dia	449	< 0,012	
3º dia	1220	< 0,012	
4º dia	2310		
5º dia	2240		
6º dia	5000		
7º dia	5000	< 0,012	111
8º dia	4030		104
13º dia	2210		
17º dia	1590		416

**Figura 2:**
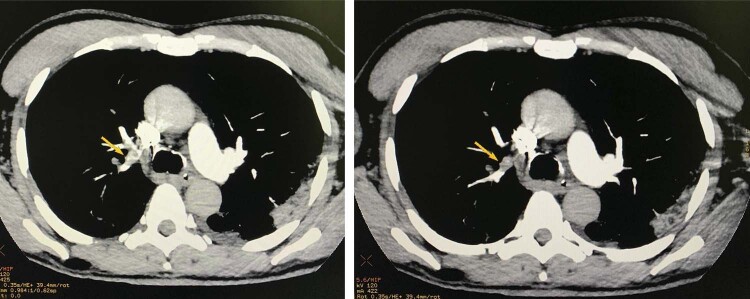
– *Angiotomografia de tórax – Seta amarela: identifica falha de enchimento na porção distal da artéria pulmonar com extensão aos ramos segmentares do lobo superior direito, compatível com tromboembolismo pulmonar.*

Diante das alterações ecocardiográficas e da significativa elevação do dímero-D (5000 ug), foi solicitado um duplex *scan* de membros inferiores, sendo o resultado negativo para trombose venosa. Foi então realizado angiotomografia do tórax ( Figura 2 ), a qual revelou falha de enchimento da porção distal da artéria pulmonar direita com extensão para os ramos segmentares do lobo superior direito compatível com tromboembolismo pulmonar. Diante desta confirmação, foi instituída anticoagulação com enoxaparina 120 mg/dia e, após 72 h, o esquema foi substituído por rivaroxabana 30 mg/dia, em decorrência da melhora hemodinâmica e programação do desmame da ventilação mecânica.

O paciente evoluiu com melhora progressiva a partir do oitavo dia de internação, coincidindo com a queda dos níveis de dímero-D, conforme visto no Gráfico 1 , sendo extubado no décimo dia de internação. Recebeu alta quatro dias após, em uso de rivaroxabana 30mg/dia por mais 17 dias e, concluídos 21 dias, 20mg/dia por três a seis meses, a ser definido após reavaliação ambulatorial.

**Gráfico 1:**
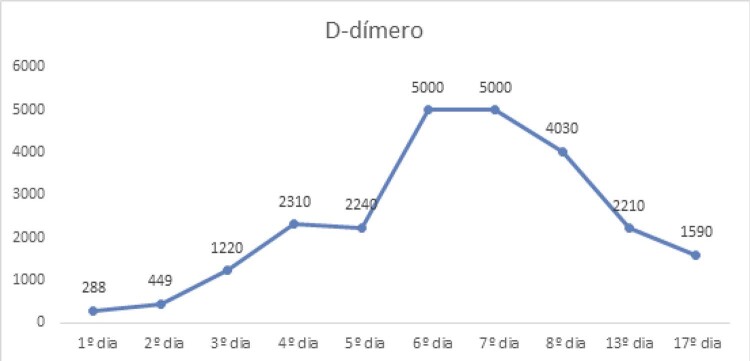
– *Curva de evolução do D-dímero durante a internação.*

## Discussão

As complicações cardiovasculares têm sido apresentadas de forma expressiva e variável na vigência da infecção pelo novo coronavirus.^5^ Na apresentação mais grave da COVID-19, observam-se altos níveis de dímero-D, que tem sido associado a aumento da mortalidade.^5^ Estudos sugerem que a resposta inflamatória sistêmica exacerbada juntamente com hipóxia possam causar disfunção endotelial e aumento da atividade pró-coagulante, contribuindo para a formação de trombos. Esse estado pró-trombótico associado à infecção sistêmica é comumente chamado de coagulopatia induzida pela sepse.^6 - 9^

Todavia, vale ressaltar que os dados disponíveis sobre risco trombótico são bastante limitados, sendo a maior parte dos eventos descritos em estudos baseados em séries de casos da China, Holanda e França.^10^ No entanto, a maioria dos especialistas concorda que o sinal para aumento do risco trombótico é suficiente para recomendar a profilaxia farmacológica do tromboembolismo venoso em pacientes hospitalizados com COVID-19. Tem sido recomendado, ainda, considerar anticoagulação no cenário de pacientes críticos em terapia intensiva, mesmo sem evidência clínica ou de imagem de trombose, levando em consideração risco de sangramento e possível benefício na interrupção da cascata pró-trombótica, segundo opinião de especialistas e série de casos, necessitando-se de estudos prospectivos para sua confirmação.^9 , 10^

A elevação do D-dímero nas formas graves da COVID-19 e a sobreposição de sintomas respiratórios da doença de base às do tromboembolismo pulmonar dificultam o diagnóstico deste último de maneira precoce. Especial atenção deve ser dada a alterações como: hipoxemia refratária, alterações eletrocardiográficas, surgimento de taquicardia sinusal não explicada pelo quadro clínico atual e disfunção de ventrículo direito para o diagnóstico de trombose pulmonar e início da terapia anticoagulante adequada.

## Conclusão

A infecção pelo SARS-Cov-2 se apresenta com um fenótipo variável, sendo frequentes os relatos de complicações cardiovasculares e a presença de um estado pró-trombótico, por mecanismos ainda não totalmente elucidados. Deve-se, portanto, ficar atento para a sobreposição das manifestações respiratórias da COVID-19, com a eventual ocorrência de embolia pulmonar, mesmo na ausência de trombose venosa profunda demonstrada. Ainda são necessários estudos para elucidação do(s) mecanismo(s) fisiopatológicos(s) das afecções tromboembólicas na COVID-19.
