# Supplementary material for: Infecção pelo SARS-Cov-2 e Tromboembolismo Pulmonar – Comportamento Pró - Trombótico da COVID-19
Source: Arq Bras Cardiol. 2020 Jul 28;115(1):142–5. [Article in Portuguese] doi: 10.36660/abc.20200427 (PMC8384314; doi:10.36660/abc.20200427)
Supplement: Translation

# Translation: SARS-Cov-2 Infection and Pulmonary Thromboembolism – The Prothrombotic State in COVID-19

**Authors:** Hellen Dutra Passos, Mariana Carvalho Alves, Leonardo Baumworcel, João Paulo Cerqueira Vieira, Juliane Dantas Seabra Garcez, Antônio Carlos Sobral Sousa

**Affiliations:** 1 Brazil Clínica e Hospital São Lucas/Rede D’Or, São Luiz, Aracaju, SE - Brazil; 2 Brazil Divisão de Cardiologia do Hospital Universitário da Universidade Federal de Sergipe, São Cristovão, Sergipe, Brazil; 3 Brazil Departamento de Medicina da Universidade Federal de Sergipe, São Cristovão, SE - Brazil; 4 Brazil Programa de Pós-Graduação em Ciências da Saúde da Universidade Federal de Sergipe, São Cristovão, Sergipe, Brazil

**Keywords:** Coronavirus, COVID-19, Pulmonary Embolism, Severe Acute Respiratory Syndrome, Anticoagulants, Diagnostic, Imaging

## Introduction

The COVID-19 caused by new coronavirus, named by the World Health Organization as Severe Acute Respiratory Syndrome CoronaVirus 2 (SARS-CoV-2), has spread all over the world with astonishing speed.^1^ In Brazil, the spread risk (R0) of COVID-19 has been 3.0, which explains its rapid dissemination all over the states.^2^

Individuals with cardiovascular diseases, systemic arterial hypertension, diabetes mellitus, chronic obstructive pulmonary disease, and immunosuppressed patients are at higher risk for adverse outcomes.^3^

A relatively high incidence of thrombotic and thromboembolic disease has been observed in COVID-19 carriers, probably due to direct effects of the SARS-CoV-2 or indirect mechanisms of the infection. Interactions between COVID-19 therapies and antiplatelet agents or anticoagulants and the inadvertent use of anticoagulants may contribute to the prothrombotic state of the disease.^4^

## Objectives

Here we describe a patient diagnosed with SARS-CoV-2 progressing with pulmonary thromboembolism and no evidence off peripheral thrombosis.

## Methods

The data here reported were obtained by review of electronic medical records, complementary tests, and literature review.

## Case Report

Patient I.M.S., male, aged 66 years, self-referred to the emergency department of a general hospital in Aracaju, Brazil, on March 28, 2020, with nasal congestion, dry cough, asthenia, nausea and fever (40^o^C) for eight days, with worsening in the last 48 hours. The patient reported having been to Rio de Janeiro for a dental implant surgery in the beginning of March and returned to Aracaju on March 18, 2020. History of osteosynthesis of the left humerus 16 years before, former smoker (quit > 10 years ago), physically active. On physical examination, the only abnormal finding was diffuse inspiratory snoring and siblings on pulmonary auscultation. The patient had normal skin color and breathing, and hemodynamically stable. The hypothesis of COVID-19 was raised and the following tests were performed: 1) laboratory routine tests, showing slightly elevated C-reactive protein levels and the other parameters within normal ranges (including myocardial injury markers); 2) RT-PCR by oropharyngeal swab; and 3) chest tomography ( Figure 1 ) which revealed ground-glass opacities, mainly in peripheral areas and more evidently in lower segments, affecting less than 50% of the pulmonary parenchyma. Electrocardiogram (ECG) with normal sinus rhythm and heart rate of 65 bpm.

**Figure 1:** – *Chest computed tomography without contrast on hospital admission. Ground-glass opacities, mainly in peripheral areas and more evidently in lower segments, affecting less than 50% of the pulmonary parenchyma.*

Since the patient was an elderly man and had impaired pulmonary function, the patient stayed in isolation of an intensive care unit, with diagnosis of viral pneumonia, probably caused by the SARS-CoV-2 infection.

A therapy with Oseltamivir (150 mg/day), Azithromycin 500 mg/day and Ceftriaxone 2g/day was initiated, combined with prophylactic enoxaparin 40mg/day for venous thrombosis. After 24 hours of hospitalization, the patient showed progressive worsening of respiratory function, culminating with acute respiratory failure, requiring orotracheal intubation on the second day of hospitalization. The patient also developed shock, and vasoactive drug was used.

With worsening of hemodynamics and the negative RT-PCR for SARS-CoV-2, a transesophageal echocardiogram (TEE) was performed to rule out the possibility of infectious endocarditis. The TEE, performed on April 02, 2020, revealed: a) enlargement of the left ventricle, which showed diffuse hypokinesis predominantly of the antero-apical wall and apical septum, grade 1 diastolic function and moderated global diastolic dysfunction, with ejection fraction of 41%; b) enlargement of the right chambers, with diffuse hypokinesis of the right ventricle, and moderate global systolic dysfunction by subjective evaluation; c) mild-to-moderate pulmonary hypertension (pulmonary artery systolic pressure of 48 mmHg); d) degenerative changes of aortic and mitral valves; e) absence of vegetations and/or thrombus. The hypothesis of myopericarditis was then raised, but both electrocardiographic findings and myocardial lesion markers were normal. Cardiac magnetic resonance imaging, which is a valuable test in these situations, was delayed due to ongoing infection.

Subsequently, an exponential increase in D-dimer and C-reactive protein levels was observed, while troponin and NT-pro-BNP levels remained within normal ranges, as described in Table 1 . It is worth pointing out that the negative RT-PCR may be explained, at least in part, by the fact that the specimens were collected on the ninth day of symptom onset, when virus release is known to be falling. Due to the high suspicion of COVID-19, the regimen of antibiotics was maintained; also, a serological test was ordered, which yielded a positive result for the disease (IgG - / IgM + SARS-Cov-2).

**Table 1:** – D-dimer, troponin, and NT-ProBNP levels during hospitalization

Hospitalization day	D-dimer (ug)	Troponin (ug/ml)	NT - ProBNP (ug/ml)
Day 1	288	<0.012	-
Day 2	449	< 0.012	
Day 3	1220	< 0.012	
Day 4	2310		
Day 5	2240		
Day 6	5000		
Day 7	5000	< 0.012	111
Day 8	4030		104
Day 13	2210		
Day 17	1590		416

Considering the echocardiographic changes and significant elevation of D-dimer (5,000 ug), a lower extremity venous duplex scan was performed to investigate venous thrombosis, and the result was negative. Computed tomography angiography of the chest was then carried out ( Figure 2 ), which revealed filling defect of the distal right pulmonary artery, extending to segmental branches of the right upper lobe, compatible with pulmonary thromboembolism. Then, an anticoagulation was initiated with enoxaparin 120 mg/day for 72 hours, substituted with rivaroxaban 30 mg/day due to improvement in hemodynamics and mechanical ventilation weaning plan.

**Figure 2:** – *Computed tomography angiography of the chest: (yellow arrow) filling defect of the distal right pulmonary artery, extending to segmental branches of the right upper lobe, compatible with pulmonary thromboembolism.*

From the eight day on, the patient showed progressive improvement with concomitant decrease in dimer-D levels, as shown in Figure 1 . The patient was extubated on the tenth day of hospitalization. Four days after, the patient was discharged, with rivaroxaban 30mg/day for further 17 days and when 21 days are completed, 20mg/day for 3-6 months, according to outpatient follow-up visits.

## Discussion

There have been remarkable and variable cardiovascular complications of the coronavirus infection.^5^ In severe manifestations of COVID-19, increased D-dimer and its association with increased mortality have been observed.^5^ Studies have suggested that an exacerbate systemic inflammatory response plus hypoxia may cause endothelial dysfunction and increased procoagulant activity, contributing to thrombus formation. This prothrombotic state, associated with systemic infection, is commonly known as sepsis-induced coagulopathy.^6 - 9^

It is worth mentioning that the data available on thrombotic risk are limited; most of the cases reported have been derived from case series in China, Holland and Fance.^10^ However, most experts agree that one sign of increased thrombotic risk is sufficient to recommend pharmacological prophylaxis of venous thromboembolism in patients hospitalized for COVD-19. In addition, anticoagulation should be considered in critically ill patients under intensive therapy, even with no clinical or imaging evidence of thrombosis, taking into consideration the risk of bleeding and potential benefit of interrupting the prothrombotic cascade, based on experts’ opinion and case series. Prospective studies are needed to confirm this benefit.^9 , 10^

The elevations in D-dimer levels in severe forms of COVD-19 and superposition of respiratory symptoms over pulmonary thromboembolism symptoms make it difficult to early diagnose the latter. Special attention must be paid to refractory hypoxemia, electrocardiographic alterations, sinus tachycardia that is not explained by current clinical condition and left ventricular dysfunction for diagnosis of pulmonary thrombosis and initiation of adequate anticoagulant therapy.

## Conclusion

The SARS-Cov-2 infection has a variable phenotype, with common manifestations of cardiovascular complications and a prothrombotic state, by mechanisms not fully elucidated. Attention should be given to superposition of respiratory symptoms of COVID-19 and eventual occurrence of pulmonary embolism, even in the absence of evidence of deep venous thrombosis. Further studies are still needed to elucidate the pathophysiological mechanisms of thromboembolic events in COVID-19.

**Graph 1:** – *D-dimer profile during hospital course.*
